# Performance of Nanotechnology in Cementitious Materials: Synthesis and Application

**DOI:** 10.3390/ma18102171

**Published:** 2025-05-08

**Authors:** Thalia Montes Rubio, Carlos Antonio Rosas Casarez, Victor Manuel Orozco Carmona, Ramiro Ahumada Cervantes, Analila Luna Valenzuela, Maria de los Angeles Cervantes Rosas, Manuel de Jesus Chinchillas Chinchillas

**Affiliations:** 1 Departamento de Ingeniería y Tecnología, Universidad Autónoma de Occidente (UAdeO), Guasave C.P. 81048, Sinaloa, Mexico; thalia_0112@hotmail.com (T.M.R.); carlos.rosas@uadeo.mx (C.A.R.C.); ramiro.ahumada@uadeo.mx (R.A.C.); analila.luna@uadeo.mx (A.L.V.); maria.cervantes@uadeo.mx (M.d.l.A.C.R.); 2 Departamento de Metalurgia e Integridad Estructural, Centro de Investigación en Materiales Avanzados (CIMAV), Chihuahua C.P. 31136, Chihuahua, Mexico

**Keywords:** nanomaterials, nanoparticles, nanofibers, concrete technology, cementitious materials

## Abstract

Cementitious materials are indispensable in the construction industry and in urban development worldwide because cement pastes, mortars, and concrete provide mechanical strength, high durability, and excellent stability to various structures that are used in a lot of civil works. Owing to the impact and relevance of these materials, it is indispensable to frequently seek ways to improve their properties and characteristics. In recent years, the development of nanomaterials such as nanoparticles (NPs) and nanofibers (NFs) has allowed cementitious materials to improve their mechanical, thermal, chemical, and durability properties, among others. This can be associated with the fact that nanomaterials allow for improved cement hydration by retaining water in the mix, helping to define a more uniform microstructure and, therefore, significantly reducing porosity, which prevents contamination such as from the entry of external agents into the structure. In addition to providing an overview of the effects of using nanomaterials on enhancing the properties of cementitious materials, this review includes the most widely used nanomaterial synthesis methods in recent years and the contribution of these nanomaterials to sustainable and environmentally friendly construction.

## 1. Introduction

Cementitious materials are considered the most important materials in modern infrastructure around the world and are causing a continuous increase in their demand [1]. These materials can be classified according to their constituent elements: (a) concrete, which is formed by mixing coarse aggregate, fine aggregate, cement, and water with or without admixtures, fibers, or other cementitious materials; (b) mortar, which is a mixture of fine aggregate, cement, and water, and may, sometimes, contain admixtures; (c) cementitious paste, which is formed from cement and water [2]. The relevance of these materials is that they are the second most used material around the world, the first being water [3]. However, the high demand generates major environmental problems such as pollution and global warming [4].

The properties of cementitious materials are very important in determining the performance, durability, and stability of civil works structures. These properties include compressive strength, adhesion, workability, and durability. However, there are also some limitations and problems in the application of these materials, including shrinkage cracking, porosity, permeability, and deterioration under aggressive conditions [5]. Therefore, it is crucial to find alternatives for improving these properties. A feasible option is the use of nanomaterials as reinforcement because it has been shown that the addition of nanoscale materials increases the properties of cementitious materials [6]. Some studies have shown that modifying the structure of cement paste at the micro and nanoscale can efficiently improve the properties and characteristics of cementitious materials [7]. Several studies focus on the application of different nanomaterials, and they evaluate their main properties, such as compressive strength, flexural strength, porosity, workability, durability, shrinkage, etcetera; however, these studies have examined the great variability in results, thus establishing the need for further studies to obtain optimal percentages for enhancing the desired properties. Below, we discuss a review of the latest works published in the field of nanomaterial synthesis while considering various methodologies. We also present the results of their incorporation into cementitious materials, demonstrating their properties and characteristics.

## 2. Nanotechnology

Nanotechnology has experienced significant growth in recent years, as it has enabled the development of materials and technology that, based on their nature, can access desired characteristics and functionalities [8]. Nanotechnology is considered a type of technology capable of creating and operating materials at the nanometric scale in a controlled way; at least one of their dimensions is at that scale. The person recognized as the father of nanotechnology is Richard Feynman, who won the Nobel Prize in Physics in 1965, thanks to his work entitled “There’s plenty of room at the bottom”, where he introduced the concept of manipulating matter at the atomic level, which created new ways of thinking based on this hypothesis [9]. There are many areas of application for this technology, for example, in medicine, in the design and use of biosensors, in the food industry, in the production of packaging, and in the pharmaceutical industry [9]. Nanotechnology has provided great benefits, positively impacting human life, mainly in the areas of health, technology, and the environment; the first two are the most impactful and recognized, primarily because they are areas with greater funding, which leads to a greater number of scientific publications on benefits and advances [10].

### 2.1. Nanomaterials and Their Morphology

**Figure 1:**
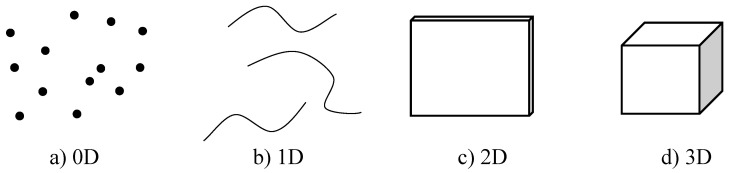
Classification of nanomaterials (design proposed by Mekuye and Abera, 2023 [13]).

A nanomaterial is a material with at least one of the dimensions of its external structure on the nanometric scale, which is between 1 and 100 nanometers [11]. Working on this scale allows new optical, thermal, magnetic, electrical, and other properties to emerge in materials, which can be useful in different fields [8]. Nanomaterials can be classified according to the number of dimensions found within the nanometric scale: 0D, when all three dimensions of the material are at the nanometric scale, such as in nanoparticles (NPs); 1D, when two of its dimensions are at that scale, such as in nanofibers (NFs); 2D, when only one of the dimensions is at the scale, such as in nanochips; and 3D, when none of its dimensions are at the nanometric scale, but its internal structure is composed by a nanostructure, such as in nanoparticle dispersions, multilayers, and nanostructured materials. This classification can be seen in Figure 1 [12].

Nanomaterials can be classified by the number of dimensions found on the nanometric scale. However, there are other classification parameters depending on their phases or manufacturing processes. These classifications can be seen in Table 1 [14].

#### 2.1.1. Characterization of Nanomaterials

The characterization of nanomaterials is important because it allows us to evaluate their shape, structure, behavior, characteristics, and properties. When materials are on a nanometric scale, it has been reported that they can enhance their performance by increasing magnetic, electrical, catalytic, optical, and mechanical properties, among others [15]. Among the characterization methods, Scanning Electron Microscopy (SEM) analysis is used to observe the surface of the material, allowing to know the characteristics of the surface, morphology, and semi-quantitative composition of the sample superficially [16]. On the other hand, Transmission Electron Microscopy (TEM) allows us to study the structure and composition of the sample, as well as the interaction between its components [17]. Additionally, Thermogravimetric Analysis (TGA) is also a highly relevant method in the study of nanomaterials since it is possible to determine the thermal characteristics and further the dynamics and kinetics of thermodegradation [18]. To understand the molecular structure and the vibration of the chemical bonds of nanomaterials, Fourier Transform Infrared Spectroscopy (FT-IR) analysis is applied; it is one of the most widely used techniques due to its ability to examine the vibrational spectra of molecules [19]. X-ray diffraction (XRD) is a technique capable of recognizing and describing the crystalline structure and properties of materials by diffracting a beam of X-rays and analyzing the angle at which this energy diffracts [20]. Another technique used for the characterization of nanomaterials is X-ray Photoelectron Spectroscopy (XPS), which is an analysis that details the properties of the surface of matter in a range of 10 nm [21]. On the other hand, Photoluminescence Spectroscopy (PL) is a characterization method that provides information on the electronic structure of materials [22]. In summary, all the characterization techniques described above are very important in nanotechnology studies because they help to understand their properties and characteristics.

#### 2.1.2. Top–Down/Bottom–Up

There is a general process for nanomaterial synthesis called top–down/bottom–up, which depends on the way of the formation process of the nanomaterial. The top–down process is based on reducing the size of bulk material into powder (nanoscale) through physical or mechanical processes [23]. These procedures have great advantages, like producing large quantities, having size and shape that can be controlled, and being applicable to many kinds of materials [24]. At the same time, they also present some disadvantages such as the generation of defects in the structures, the requirement for complex and expensive equipment or specialized techniques, and the possibility of polluting the sample in the mechanical process [25]. Instead, the method known as “bottom–up” tries to build the material through a chemical process from the union of atoms, ions, or individual molecules until forming clusters (nanomaterials) [26]. Some advantages of this procedure include greater control over the structure and final composition of the nanomaterial, fewer defects, and less contamination, and the structures can be varied to change the properties of the materials. Some disadvantages include the need for very specific controlled conditions (temperature, pressure, purity, humidity, among others); it is difficult to produce nanomaterials in large quantities; some methods can be slow; require sophisticated and expensive equipment, also dangerous reagents that can damage the environment [27].

### 2.2. Synthesis of Nanoparticles

Nanoparticles (NPs) are dispersions of particles or bodies whose three dimensions are on the nanometric scale. Some authors mention that the nanometric scale ranges from 1 to 100 nm. Refs. [28,29,30] and others mention that this scale is between 10 and 1000 nm [31,32,33]. The process of NPs synthesis took place between the 14th and 13th centuries B.C. when the Egyptian and Mesopotamian cultures carried out the process of manufacturing glass by adding metal oxides [34]; therefore, harnessing the properties of materials at this scale has been applied for hundreds of years. Converting a material to the nanoscale increases specific properties that can improve its performance in different applications, for example, an increase in surface area, antibacterial properties, hardness, electron band gap, and electrical and magnetic properties, among others [35]. On the other hand, NPs can be classified according to their physical and chemical properties into carbon-based, metallic, ceramic, semiconductor, polymeric, and lipid-based [36].

Nowadays, there are different physical and chemical methods for NPs synthesis. Some physical processes include milling, lithography, arc discharge, and laser ablation, etc. [27]. These methods do not need specialized chemical reagents, and they also allow a fast and efficient production of NPs with a high purity. These advantages make these processes widely used for some industrial applications.

Furthermore, some chemical methods are chemical reduction, photochemical synthesis, and sol-gel, among others [37], which achieve synthesize NPs from a variety of materials; they also allow for more detailed control of the shape and size and the creation of NPs with better properties and characteristics by functionalizing molecules of different nature [38]. These methods are used in different applications like the generation of sensors [39], electronics [40], catalysis [41], biomedicine [42], etc. Within these chemical processes, the biological method has been relevant in recent years, which is based on the use of natural elements like plants, leaves, stems or fungi for the formation of NPs, this method is known as “green synthesis” [43].

#### 2.2.1. Lithography

It is known as a procedure for modeling semiconductor material capable of achieving this on nanometric scales through the printing of desired shapes and structures by eliminating specific areas in light-sensitive material [44]; some of the most commonly used techniques are e-beam, X-ray, EUV, Ion-beam and optical lithography [45]. The main advantage of this synthesis method is its ability to produce anything from a single nanoparticle to a group of nanoparticles with the desired properties. However, the main limitation is the requirement for sophisticated and often expensive equipment [26]. In 2021, Chiang et al. used the NP chemical lift-off lithography technique to pattern a monolayer of gold NPs through a polydimethylsiloxane (PDMS) patterning mold; this was achieved due to the breaking of weak Van der Waals bonds between molecules, and the purpose of the study was to use it in the manufacture of building blocks [46]. At the same time, McGrath et al., 2021 proposed a procedure for the fabrication of silver grids by creating a pattern using silver (Ag) NPs ink with the nanoimprint lithography method; these grids had a good performance with this technique, which is simpler and could facilitate roll-to-roll processes [47]. Also, Miura et al., 2023, created a gas permeable mold using the nanoimprint lithography technique to manufacture biomimetic antibacterial nanostructures by the microinjection method for mass production, with the aim of evaluating their antibacterial activity, which showed promising results [48].

#### 2.2.2. Laser Ablation

**Figure 2:**
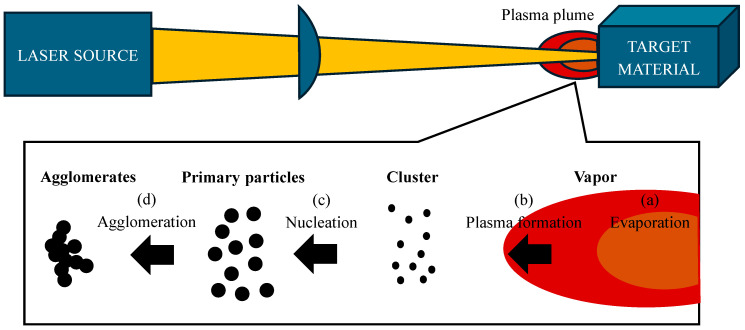
Laser abrasion synthesis (design proposed by Kim et al., 2017 [50]).

Laser ablation works by means of an electrically powered laser and achieves the ablation of a solid object, which must be in a liquid or gaseous environment. This process produces NPs in powder or colloidal form, as shown in Figure 2 [49]. One of the main advantages of this method is the high purity of the NPs obtained because there is no contamination of the reactor due to the purity of the material and the environmental conditions. However, the disadvantage is that it presents little control over the size, shape, crystalline structure, and agglomerations because the formation of NPs by this method is related to the Brownian motion of the molecules [50]. This method has achieved relevance due to its speed and the wide variety of NPs that can be generated due to their metallic, semiconductor, and polymeric nature [44]. In 2021, Rashid et al. synthesized pure gold (Au) and zinc (Zn) NPs separately, as well as core–shell NPs of both elements by the laser ablation method; the authors mention that the particle size increases when the laser energy also increases; also the NPs presented a hemispherical shape and a different size distribution [51]. In the same year, Khashan et al. used the same method but in the liquid phase for the formation of titanium dioxide (TiO_2_) NPs. In the study, the presence of flat crystalline phases, a reduction in size distribution with increasing ablation time, and a spherical structure was obtained; additionally, its antibacterial activity was evaluated, having a good result against *E. coli* [52]. Furthermore, in 2022, Elsayed et al. developed colloidal bimetallic NPs of zinc oxide and silver (ZnO-Ag) with laser ablation, with the aim of evaluating their anticancer activity, resulting in an adequate performance [53].

#### 2.2.3. Mechanical Milling

**Figure 3:**
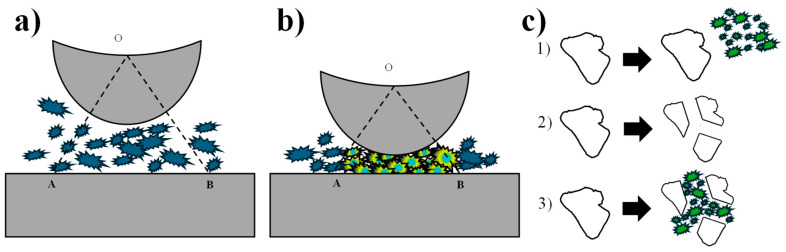
Synthesis process by mechanical milling: (**a**) synthesis process without impact; (**b**) impact at a moment of maximum force; (**c**) process of material fragmentation (design proposed by Gorrasi and Sorrentino, 2015 [54]).

Mechanical milling is an environmentally friendly option for nanomaterial synthesis. It involves plastic deformation through the application of repeated high-impact force, with the purpose of reducing particle size and mixing them into new phases (Figure 3) [54]. This method takes as reference three mechanisms of fracture of the materials: (1) abrasion, when force is applied at low intensity obtaining small particles that come off the original material; (2) cleavage, occurs when a force is applied to the material with greater intensity but slowly, generating particles of similar size; (3) fragmentation, occurs when loads are applied intensely in short periods of time, thus generating the division of the material into a wide range of sizes as shown in Figure 3c [54]. Some of the equipment used in this methodology are vibratory mills, planetary ball mills, and attritor mills [55]. Some parameters that may vary in this method according to the equipment or material to be tested are the amount of material, time, grinding speed, and ball size [56,57]. In 2021, Shojaei et al. synthesized Cu, Al, and S NPs for two hours using a high-energy ball mill; their research demonstrated a decrease in crystallite size and an increase in microdeformation with increasing milling time [57]. In the same year, Velásquez and Urquijo synthesized magnetite-maghemite NPs in the presence of polyethylene glycol as a stabilizing medium during a 24 h milling period. The characterization demonstrated high purity of magnetite and maghemite, a nearly spherical shape, and an average particle size of 14 nm. The authors conclude that the mechanical milling method is efficient for obtaining nanoparticles from these materials [58]. Using the ball milling method, Wirunchit et al., 2021, synthesized zinc oxide (ZnO) NPs at different calcination temperatures; the characterization showed a spherical morphology, and the smallest particle size was obtained at 800 °C. These NPs were biologically evaluated, resulting in a high inhibitory effect on bacteria [59].

#### 2.2.4. Sol-Gel

**Figure 4:**
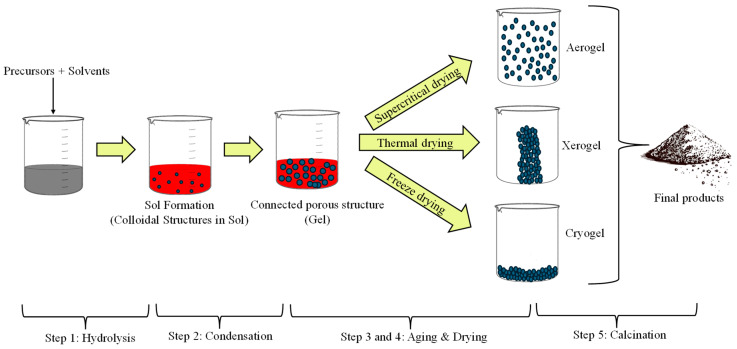
Synthesis of NPs by sol-gel method (design proposed by Bokov et al., 2021 [61]).

This nanomaterial synthesis procedure involves the preparation of inorganic polymers and ceramics through irreversible chemical reactions in solutions. This is due to the transformation of liquid precursors into a “sol” through hydrolysis and condensation of metal alkoxide precursors, to eventually become a network structure known as a “gel” [60] (see Figure 4). This method offers several advantages that make it one of the most widely used and oldest. Some of these advantages include its simple process, the ability to obtain high-purity products, high and efficient production, surface coverage, modification of physical properties (like a decrease in the coefficient of thermal expansion and UV absorption), increased optical transparency, low initial investment with high-quality results, etc. [61]. Some of the metal oxides used in this method for NPs synthesis are ZnO, TiO_2_, tungsten oxide (WO_3_), and tin oxide (SnO_2_); some of their applications are photocatalysis, photovoltaic cells, gas detection, hydrogen fuel production, biomedical and energy storage [62].

In 2024, Eslami et al. synthesized magnesium aluminate (MgAlO) spinel NPs by the sol-gel method with stearic acid as a capping agent; this process resulted in an average particle size of 12 nm, good hydrogen storage capacity, and exceptional electrochemical performance; the authors consider the material a valuable advancement in hydrogen storage technology [63]. In the same year, Kistan et al. synthesized manganese (Mn) doped ZnO NPs using the same method to evaluate their photocatalytic activity for use in the removal of organic pollutants and wastewater; the characterization showed a high degree of crystallinity, a spherical morphology with an average particle size of 20 nm, and excellent photocatalytic performance, making them a potential wastewater treatment [64]. Additionally, Renganathan et al. synthesized copper oxide (CuO) NPs and deposited them on polymethylmethacrylate (PMMA) fibers to use them as an ammonia gas detector; this experimentation had a superior performance in the detection of ammonia due to it being more sensitive to this gas and having a faster response and recovery rate at room temperature compared to the detection of ethanol and methanol [65].

#### 2.2.5. Biosynthesis

**Figure 5:**
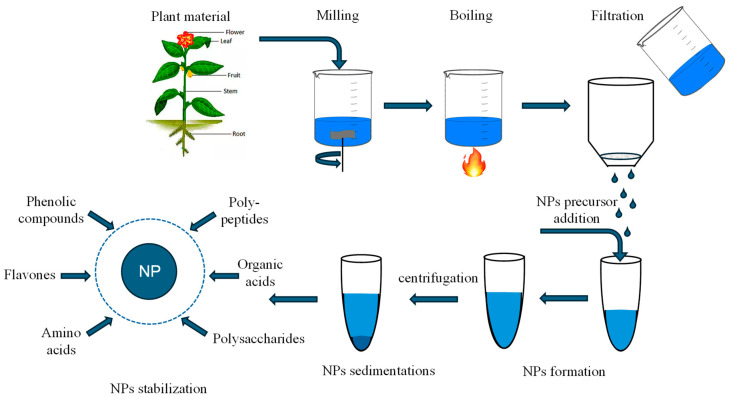
Synthesis of NPs by bio synthesis method (design proposed by Chopra et al., 2022 [67]).

This method has been recognized for its green and environmentally friendly technology for the reason that it is produced from microorganisms such as plants, bacteria, actinomycetes, yeast, algae, fungi, etc. [66]. NPs can only be obtained from certain organisms, depending on their enzymatic activity and metabolic processes. These organisms are capable of collecting inorganic metal ions from their environment, which promotes this activity [67]. According to their characteristics such as shape, size, structure, and physical, chemical, and biological properties, NPs obtained by biosynthesis have different fields of application like biomedicine [68], drugs [69], cancer therapy [70], food and agriculture [71], food packaging [72], pesticides [73], water treatment [74], pollution monitoring sensors, UV protection [75], etc. This method begins with the collection of the organism or plant of interest, where it must be washed with distilled water to remove impurities, then it is left to dry and crushed until it becomes powder; subsequently, the extract is obtained and a precursor is added to be stirred, in such a way that the chemical process begins; then high temperatures are applied until the NPs are obtained [76]. This process is shown in Figure 5.

In 2024, Albert et al. performed a biosynthesis of Ag NPs, which obtained an average particle size of 23 nm, resulting in a higher rate of surface area/volume; as a result, these NPs inhibit the growth of bacteria, both beneficial and dangerous to humans, making them a potential material for use as a coating on surgical equipment for aseptic operators in the healthcare industry [77]. Ohiduzzaman also performed biosynthesis of Ag NPs but with banana pulp extract, which obtained an average particle size of 42.97 nm; subsequently, they evaluated their performance in their antibacterial activity with *E. coli* and *S. epidermidis*, as well as their catalytic effect to improve the performance of electrochemical cells; thus, it is concluded that these NPs have excellent biomedical applications and can help in the creation of economic and ecological devices for the generation of electrical energy [78]. Further, Abdel-Maksoud obtained TiO_2_ NPs through biosynthesis for use in the biocontrol of different strains of isolated fungi. A characterization showed a spherical morphology, a particle size of 3–7 nm, and low toxicity at high concentrations. This resulted in satisfactory performance in inhibiting the growth of different fungal strains [79].

### 2.3. Synthesis of Nanofibers

Nanofibers (NFs) are a kind of nanomaterials where two of their dimensions are on the nanometric scale, with their third dimension, the length, being significantly greater compared to the other two dimensions of their cross section [80] when the fiber diameter reaches the nanometric scale, notable enhancements in properties are observed, including an increase in the surface area to volume rate, greater flexibility in the surface functionalities, and a significant improvement of its mechanical properties [81]. Currently, there are many methods to synthesize these nanomaterials, including drawing, template synthesis, self-assembly, phase separation, melt blowing, electrospinning, and blow spinning [82,83]; each of them has different advantages and disadvantages, but the last three (melt blowing, electrospinning and blow spinning) are methods with very interesting characteristics since they allow the production of a large number of NFs at low cost, with good thermal and mechanical properties, as well as being environmentally friendly [84].

There are different methods to synthetize NFs, which, despite having the objective of developing NFs, can change according to certain properties such as fiber diameter, orientation, porosity, agglomeration, defects, and resistance, among others. Each of them is based on different application techniques and mechanisms, such as chemical procedures, high airflow, electricity, or heat. Next, some methods used in the synthesis of NFs will be analyzed, such as Centrifugal jet spinning, CO_2_ laser supersonic drawing, melt spinning, electrospinning, and blow spinning.

#### 2.3.1. Centrifugal Jet Spinning

**Figure 6:**
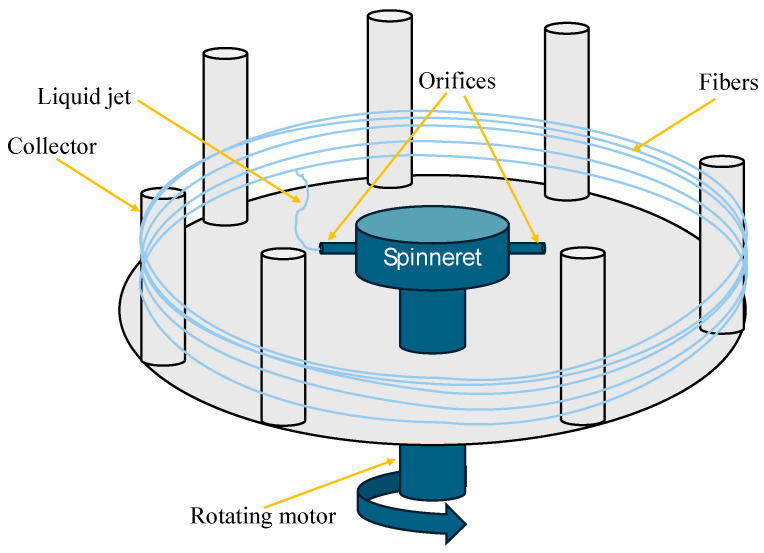
Synthesis of NFs by centrifugal jet spinning method (design proposed by Gholipour-Kanani and Daneshi, 2022 [86]).

This method, also known as Rotary jet spinning, was proposed in 2010 by Badrossamay et al. It is based on the use of a nozzle capable of rotating at high speeds through which a constant flow of polymer solution would be expelled and subjected to centrifugal force, getting stretched before solidifying [85]. The schematic of this process is illustrated in Figure 6. Centrifugal jet spinning has been patented, being the trademark of the first machine called Forcespinning TM by FibRio^®^ Technology Co., McAllen, TX, USA [86]. Some of the polymer solutions used in this method are Polyvinylpyrrolidone (PVP) dissolved in 10% Ethanol [87], Poly-L-lactic acid (PLLA) dissolved in 10% Methylene Chloride [88], PVP dissolved in 4% acetic acid, 9% Polyvinyl alcohol (PVA) and 4% Polyethylene oxide (PEO) both dissolved in water [89], among others. It is important to highlight that the morphology of the resulting NFs in this method highly depends on centrifugal force and viscous force. The first is related to the dimensions of the rotating head and the rotation speed; the second is related to the molecular weight of the polymer and the concentration of the polymer solution [90].

Jingying Xu et al., 2024, created two fibrous membranes using the centrifugal jet spinning method: one of them using Polyacrylonitrile (PAN) with ZnO NPs and the other with PAN and Polyvinylidene fluoride (PVDF). The application of these membranes was to use them in the photocatalytic degradation of contaminants present in water, achieving great results [91]. Meanwhile, Yaru Wang et al. synthesized PVDF NFs using the same method; the main objective was to use these NFs as oil–water separators; synthesis procedure allowed to obtain pure PVDF NFs with some nano striped protrusions on the surface, high specific surface area, and a rough morphology [92]. Simultaneously, Mei et al. used the same method to synthesize thermoplastic Polyurethane (TPU) NFs with the aim of creating a conductive hybrid film using NFs and carbon nanotubes as conductive fillers by ultrasonic dispersion and printing techniques. The film showed excellent results in electrical conductivity (31.4 S/m), elongation at break (217.5%), and stability in the tension cycle (800 cycles at 20% tension). In addition, tests were conducted adhering these films to the human body to verify their potential as flexible sensors in the changes in movement, which presented excellent performance in movements of wrists, elbows, and thoracic cavities [93].

#### 2.3.2. CO_2_ Laser Supersonic Drawing

**Figure 7:**
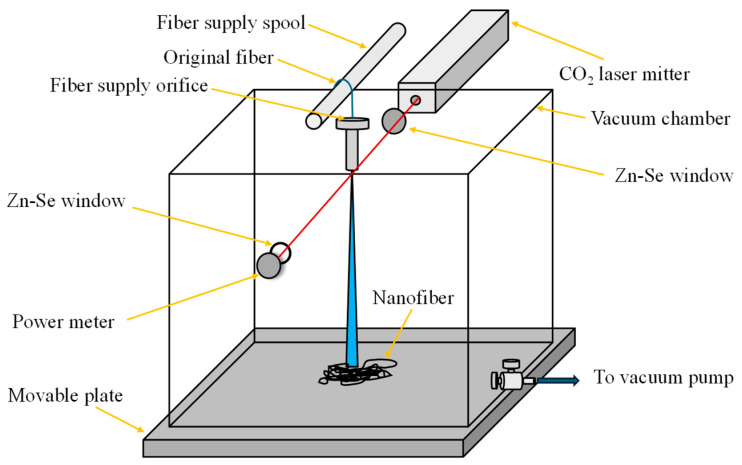
Synthesis of NFs by CO_2_ laser supersonic drawing method (design proposed by Koyama et al., 2014 [95]).

The main characteristic of this technique is the absence of chemical solvents for obtaining NF. This procedure works through a CO_2_ laser, which melts fibers with diameters of 100 to 200 μm and then passes them through a supersonic airflow, allowing their elongation and generating a diameter on the nanometric scale (see Figure 7) [94]. There are various parameters that must be considered to obtain a better morphology of the NFs; one of the most evident is the relationship between the pressure of the chamber and the diameter of the resulting NFs [95]. It is considered novel due to its simplicity because it only requires CO2 irradiation, in addition to not requiring chemicals or additional processes. This makes it a process applicable mainly in medicine since it does not require additional substances that can be harmful to the human body [96].

Suzuki and Oshiro, 2021, created Polyethylene Naphthalate (PEN) NFs using the CO_2_ laser supersonic drawing method and employing a low-temperature supersonic laser, which was directed into a vacuum chamber. Various tests were performed, and nanofibers with the smallest diameter (0.249 nm) were obtained by applying a laser power of 4 W and a chamber pressure of −98 kPa, achieving an increase in the melting temperature from 260 to 285 °C. This was attributed to the characteristic supramolecular sequence effect at the nanometric scale. In addition, the authors denote the importance of this method in its ability to form NFs without the need for a solvent or the elimination of other components [97]. Furthermore, 2020, Ueta et al. synthesized Polyethylene terephthalate (PET) NFs using a CO_2_ laser of 4W in a supersonic flow, obtaining NFs with an average diameter of 500 nm and a smooth surface morphology. The resulting NFs were used to form a sheet to be used in an extraction capillary for the determination of Polycyclic aromatic hydrocarbons in different water samples. The results showed satisfactory performance due to the ease of avoiding the analyte extracts of this artifact for the low porosity of the NFs and their greater surface area [98]. In 2017, Koyama et al. also implemented this technique for the manufacture of Polyphenylene sulfide (PPS) NFs sheets; in this procedure, there were applied eight holes for the supply of the polymer to form the NFs sheets, with an average diameter of 700 nm [99].

#### 2.3.3. Melt Spinning

**Figure 8:**
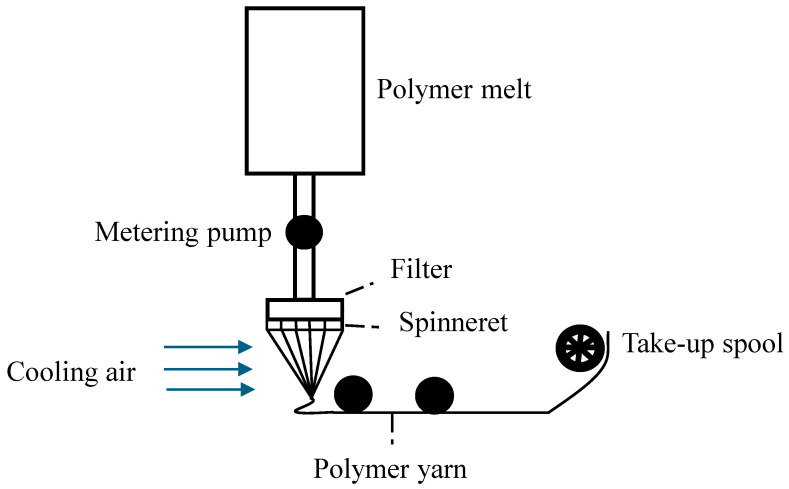
Synthesis of NFs by melt spinning method (design proposed by Qu and Skorobogatiy, 2015 [105]).

This procedure is defined as a one-step method in which high velocity fluid streams (typically air) act upon polymer solutions subjected to elevated temperatures from an extruder nozzle to a collector, as illustrated in Figure 8 [100]. This technique was developed in 1960 through work with metastable solid solutions [101]. The method involves several variables that influence the characteristics and properties of the resulting nanofibers, such as the type of material, fluid dynamics, air pressure, solution temperature, and process temperature (i.e., from the extruder nozzle to the collector). Some researchers have proposed the use of digital simulators and mathematical models to estimate the resulting fiber properties produced by melt spinning. This approach would allow for the optimal configuration of each process parameter to obtain fibers with desired properties depending on their intended application [102].

In 2023, González Sánchez et al. used this technique to fabricate textile fiber prototypes from a polyester fiber nanocomposite reinforced with CuO nanoparticles. The objective was to evaluate their performance against pathogenic organisms such as *S. aureus*, *E. coli*, and *C. albicans*. The results showed excellent antimicrobial performance against the first two pathogens, achieving inhibition rates greater than 99% [103]. In a separate study, Ji et al. (2022) employed melt spinning to produce a composite fiber membrane composed of polyvinylidene fluoride (PVDF), calcium chloride (CaCl_2_), and polyethylene glycol (PEG). The aim was to achieve a highly porous structure suitable for oil–water separation applications. The best results were obtained with higher PEG content. Furthermore, the process was considered environmentally friendly because it does not require solvents or diluting agents [104].

#### 2.3.4. Blow Spinning

**Figure 9:**
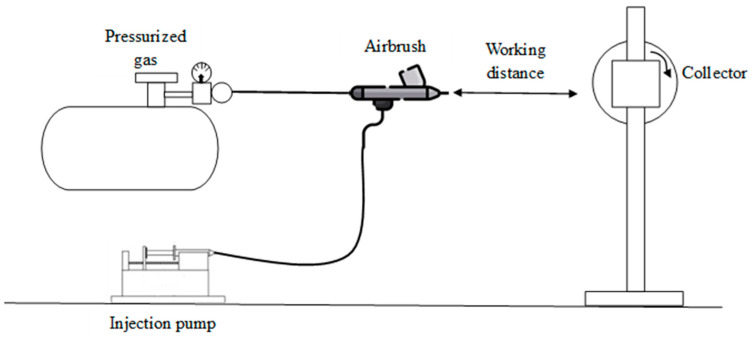
Synthesis of NFs by blow spinning method (design proposed by Medeiros et al., 2009 [108]).

Blow spinning is a nanofiber (NF) fabrication method based on the application of concentric and parallel streams of pressurized air to a polymer solution, which must consist of a polymer dissolved in a volatile solvent [106]. The configuration of this technique is illustrated in Figure 9. This method combines the principles of electrospinning and melt blow spinning, offering several advantages such as high production efficiency, shorter preparation time, simple equipment requirements, no need for polymer conductivity, enhanced safety, and relatively low cost [107]. The technique was first proposed by Medeiros, 2009 [108], who successfully obtained micro- and nanofibers by adjusting various parameters such as working distance, injection rate, gas flow pressure, and polymer concentration—all of which significantly influenced fiber morphology and production rate. One of the key advantages of this method is its electricity-free operation, making it easier and safer to use, particularly in medical contexts. For instance, in the medical field, this method has been studied for its application in scaffold fabrication for tissue regeneration, due to its practicality in surgical settings [109].

In 2021, Chen et al. developed a nanocomposite using SnO_2_ nanoparticles and nanorods combined with carbon nanotubes via the blow spinning method. These materials subsequently underwent a self-assembly process involving pre-oxidation and high-temperature calcination, using SnCl_2_ and PAN as precursors. The resulting nanocomposite exhibited outstanding electrochemical properties, making it a promising candidate for use as electrode materials in electric vehicles and household appliances [110]. In the same year, Du et al. synthesized nanofibers from ZnO nanoparticles and PAN to exploit their photocatalytic and antibacterial properties. The composite demonstrated excellent performance in the photodegradation of organic dyes, achieving a degradation rate of 94–98% after five cycles, and inhibition rates above 99% for *E. coli* and *S. aureus.* These results indicate the potential of the nanocomposite for applications in wastewater treatment and antibacterial [111]. Additionally, Sardareh et al. (2022) synthesized a nanocomposite composed of gelatin and polylactic acid (PLA) nanofibers loaded with gold nanoparticles (Au NPs) using the blow spinning method, which was selected for its energy efficiency and high productivity. The resulting nanocomposite had an average fiber diameter of 335 nm. The authors evaluated its performance as a wound dressing, and the results revealed significant antibacterial activity against both Gram-positive and Gram-negative bacteria, along with low cytotoxicity [112].

#### 2.3.5. Electrospinning

**Figure 10:**
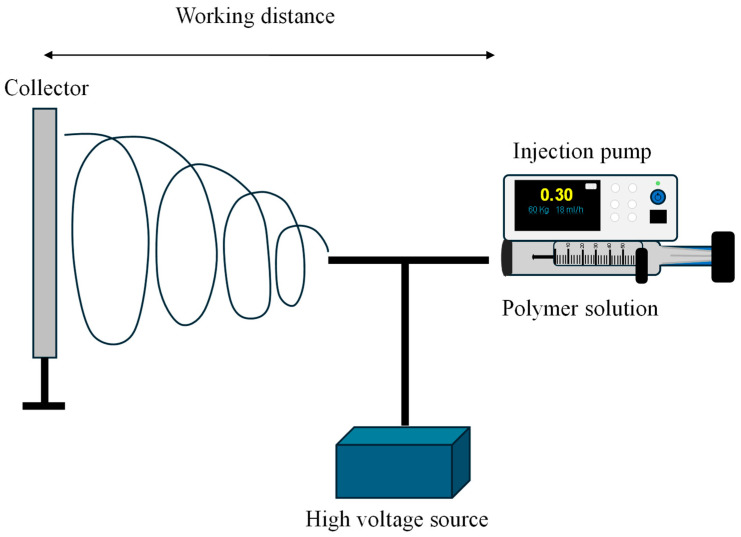
Synthesis of NFs by electrospinnig method (design proposed by Alharbi et al., 2016 [125]).

Electrospinning is one of the most widely used techniques in recent years for the fabrication of nanofibers (NFs). By varying the parameters of this method, it is possible to control the diameter of the resulting fibers since the polymer solution is manipulated using electrostatic forces, as illustrated in Figure 10 [113]. Currently, this method enables the formation of fibers ranging from 2 nm to the micrometer scale and can utilize both natural and synthetic polymers [114]. The origins of this fiber production method date back to the early 20th century. In 1902, Cooley and Morton filed the first patents for devices related to the behavior of dielectric liquids under electric charge [115,116]. Building on this, Anton Formhals developed patents for equipment and processes aimed at fiber fabrication through electric fields [117,118,119]. Through this method, polymeric NFs with various mechanical and chemical properties have been produced, enabling their optimal structure and morphology for a wide range of applications, including optical fibers, filtration devices, drug delivery systems, textiles, tissue scaffolds, and more [120]. While electrospinning has been adopted in multiple industries, particularly textiles, it faces significant challenges, primarily due to its relatively low production rate and the difficulty of achieving controlled and uniform fiber alignment compared to other techniques [121].

Nowadays, a wide range of research employs electrospinning. For instance, in 2024, Gong et al. proposed the fabrication of core–shell NFs, where the core consisted of polyethylene oxide (PEO), and the shell was composed of PEO, poly (vinyl alcohol-co-ethylene) (PVA-co-PE), and the antibacterial agent vitamin K3 (VK_3_). The resulting fibers exhibited an average diameter of 289.8 nm for the core–shell structure and 53.5 nm for the core, demonstrating good antibacterial performance against *E. coli* and *S. aureus*, along with favorable tensile strength and toughness [122]. Similarly, Dong et al. (2024) developed core–shell NFs using this method to control drug release profiles over time. They employed a water-soluble polymer (PVP) and a water-insoluble one (ethylcellulose, EC) to modulate the controlled-release behavior of ferulic acid (FA) as the active pharmaceutical ingredient. The fibers showed smooth morphology without beading or defects, attributed to good compatibility between the polymers and the synthesis method. In vitro dissolution tests demonstrated that both EC and PVP contributed to effective FA release control, primarily influenced by the water permeability rates associated with each polymer’s solubility [123].

In 2022, Alshaya et al. synthesized NFs via electrospinning for oral delivery of nifedipine and atorvastatin calcium, which are two drugs commonly prescribed for hypertension and hyperlipidemia. The nanofibers exhibited a smooth surface, free of beads or pores, with an average diameter of 968 ± 198 nm. Drug release profiles showed 61% and 47% release of nifedipine and atorvastatin, respectively, within the first 10 min, with complete release achieved within 120 min. This innovative approach offers a promising option for oral drug delivery systems aimed at improving patient compliance, particularly in geriatric populations [124].

## 3. Nanotechnology Applied to Cementitious Materials

The construction industry is a fundamental pillar of urbanization, and it continues to grow due to the increase in global population. According to reports from the United Nations, it is projected that nearly 70% of the world’s population will reside in urban areas by 2050 [126], underscoring the global significance of this sector. However, the construction industry faces numerous challenges that impact its efficiency, quality, and environmental sustainability. One of the most concerning issues is the need for cement and aggregates in virtually all civil engineering projects. Cement production is associated with significant environmental pollution, and the extraction of natural aggregates causes problems in ecosystems [127,128]. In response, multiple research efforts aim to mitigate these problems and transition towards more sustainable or “green” construction practices. Moreover, it is essential for cementitious materials employed in this industry to meet specific strength and durability criteria. To address this, some studies focus on enhancing these properties using additives, chemical admixture materials added in small quantities before or during the manufacturing process to improve specific functions; and additions, which are incorporated to enhance the mechanical and durability properties of cementitious composites [129]. Both approaches are investigated to ensure higher quality and longer-lasting construction outputs.

In recent years, the number of studies exploring the application of nanotechnology to improve cementitious materials has notably increased. The incorporation of nanomaterials has been shown to reduce porosity, thereby increasing durability and improving resistance to external agents such as moisture and chemicals [130,131]; furthermore, they contribute to enhanced thermal properties and significantly reduce water evaporation, promoting a more homogeneous setting process and reducing shrinkage in cement-based materials [132]. The use of nanomaterials, such as NPs and NFs, represents a promising area of innovation that facilitates the development of more durable, higher-quality, and environmentally friendly construction materials.

### 3.1. Mechanical Properties

One of the main properties of cementitious materials is their compressive and flexural strength, as these characteristics are essential for their applications within the construction sector. Table 2 compiles recent research studies in which nanomaterials were incorporated into cement-based materials, such as concrete and mortar, with the aim of improving their mechanical and durability properties. Most of the studies included in the table used small amounts of nanomaterials and maintained a relatively low water-to-cement (w/c) ratio. The results for compressive and flexural strength are expressed as percentages relative to the control sample used in each study. A positive sign indicates an increase in the property, whereas a negative sign denotes a reduction.

Among the nanomaterials used to enhance mechanical properties, silicon dioxide (SiO_2_) nanoparticles are particularly notable. This material has attracted considerable attention due to its controllable particle size, low toxicity, simplicity, stability, and ease of integration with other materials [133]. Table 2 presents various studies utilizing SiO_2_ NPs to evaluate improvements in compressive and flexural strength. These studies report particle sizes ranging from 10 to 30 nm and w/c ratios between 0.4 and 0.55. An exception is a study conducted by Palla, R., 2017, in which SiO_2_ NPs and fly ash were added as a cement substitute (w/c: 0.25) [134]; the authors concluded that the inclusion of these NPs significantly improves the mechanical properties of cementitious materials. They also noted that the concentration of nanomaterials should not exceed 2% of the cement weight, as higher concentrations can reduce the mixture’s workability due to decreased fluidity [135]. Additionally, irregularly shaped nanomaterials increase the specific surface area, which in turn raises water demand during concrete preparation [136].

Carbon nanotubes (CNTs) are another widely studied nanomaterial for enhancing mechanical performance, owing to their exceptional mechanical strength, hardness, and thermal properties; these characteristics make CNTs promising candidates for incorporation into cementitious composites [137]. Several studies have demonstrated that low CNT concentrations (0.1–0.5%) can produce significant improvements, with compressive strength increasing by 18–23% and flexural strength by approximately 10–22% [138,139,140]. Moreover, other nanoparticles such as titanium dioxide (TiO_2_), aluminum oxide (Al_2_O_3_), and ferric oxide (Fe_2_O_3_) have shown compressive strength increases ranging from 5% to 25% and flexural strength improvements from 3% to 45% [136,141,142].

Nanofibers (NFs) have also significantly contributed to improving the properties of cementitious materials. For example, Li et al. (2024) investigated the performance of concrete reinforced with hybrid NFs composed of carbon black (commonly known as soot) and charcoal. Their results indicated that the best performance was achieved with 0.5% carbon black NFs and 0.2% charcoal NFs, attributing the improvement in mechanical properties to the NFs’ ability to fill voids and reduce porosity [143]. In the same year, Zhang et al. conducted a review of different NFs used in cementitious systems, concluding that carbon-based NFs are predominant in this field. They emphasized that, with proper dosage, these NFs can improve both mechanical properties and durability by preventing crack formation at the nanoscale level [144]. An additional important aspect to consider is the economic and environmental impact of using nanomaterials in cementitious materials. In this context, Rajendran et al. (2025) evaluated the economic and environmental performance of concrete reinforced with cellulose NFs compared to traditional concrete. Their findings reported that both the cost and environmental impact of producing 1 m^3^ of NF-reinforced concrete were lower than those associated with conventional concrete [145].

**Table 2:** Mechanical properties of cementitious materials with the addition of nanomaterials.

Nanomateriales en Materiales Cementantes	Size	w/c	%	F’C	M.R.	Reference
Carbon Nanotubes in mortar	8–15 nm	0.4	0.10%	18.40%	22.23%	[140]
Cellulose NPs in mortar	5–100 nm	0.4	0.50%	23.40%	30.46%	[140]
SiO_2_ NPs in mortar	20–30 nm	0.4	2%	23.09%	14.91%	[140]
Carbon Nanotubes in concrete	>50 nm	0.55	0.10%	22.30%	NE	[138]
Carbon NPs in concrete	150 nm	0.4	1.20%	1.00%	NE	[146]
SiO_2_ NPs in concrete	15–20 nm	0.4	0.60%	17.58%	NE	[146]
(SiO_2_ + Ca) NPs in concrete	Two previous	0.45	0.4% NC + 0.6 NS	9.16%	NE	[146]
Fe_2_O_3_ NPs in concrete	15 nm	0.4	1.00%	15.49%	NE	[142]
SiO_2_ NPs + fly ash in concrete	116 m^2^/g *	0.25	2%	30.00%	23%	[134]
SiO_2_ NPs in concrete	200 m^2^/g *	0.4	2%	15.00%	NE	[147]
Al_2_Si_2_O_5_(OH)_4_ (Halloysite) Nanotubes in concrete	29 m^2^/g *	0.4	3%	13.10%	NE	[147]
Al_2_H_2_O_12_Si_4_ (Montmorillonite) NPs in concrete	27 m^2^/g *	0.4	3%	23.10%	NE	[147]
SiO_2_ NPs in concrete	10–20 nm	0.42	4%	7.90%	2.80%	[136]
TiO_2_ NPs in concrete	<25 nm	0.42	2%	2.70%	3.90%	[136]
Al_2_O_3_ NPs in concrete	<50 nm	0.42	0.50%	4.50%	−2.20%	[136]
Carbon Nanotubes in concrete	149 nm	0.2	0.50%	14.20%	10.40%	[139]
SiO2 NPs in concrete	15 nm	0.4	4%	NE	45%	[141]

F’C: Compressive strength; M.R. Flexural strength; NE: Not evaluated; * Specific Surface area.

### 3.2. Durability

The durability of cementitious materials is a critical factor in the construction industry, as it determines both the service life and the performance of these materials when exposed to aggressive or adverse environments [148]. Durability encompasses the capacity of cementitious materials to withstand physical, chemical, or biological agents that may lead to their deterioration [149]. Therefore, all construction works should regard durability-related properties as essential design and performance criteria.

The application of nanomaterials in construction has emerged as an innovative strategy to enhance specific properties of cement-based materials. Certain nanomaterials contribute to reduced porosity in cementitious matrices, thereby enhancing mechanical performance and offering protection against degradation caused by external agents [150]. Consequently, researchers worldwide are investigating the effects of nanomaterial incorporation on improving the durability of cementitious systems.

In 2024, Maohua Zhang et al. examined chloride ion resistance and freeze–thaw cycling performance of marine concrete modified with SiO_2_ and Fe_2_O_3_ nanoparticles using a 5% NaCl solution. The optimal nanoparticle dosage was found to be approximately 5%. The researchers concluded that the addition of these nanoparticles accelerates the formation of hydration products and enhances both the chemical bonding capacity and the physical adsorption capability of the hydrated products to Cl^−^ ions. Moreover, the incorporation of these nanoparticles significantly reduced the free Cl^−^ ion content in the concrete matrix [151]. In a separate study, Radhika Sridhar et al. (2024) incorporated 2% nanoalumina (Al_2_O_3_) and 0.3% polyvinyl alcohol (PVA) nanofibers into cement-based concretes and mortars. They assessed mechanical and microstructural properties, as well as rapid chloride permeability test (RCPT). The results obtained in 28 days showed substantial improvements in durability, attributed to the small effective particle size and high specific surface area of the nanomaterials, which promoted beneficial chemical reactions with other compounds [152]. By contrast, Abiola Usman Adebanjo et al. (2024) incorporated 2% TiO_2_ and 1.33% ZnO nanoparticles into a high-performance concrete matrix. Although mechanical properties improved, no enhancement in durability was observed. The specimens were exposed for three months to a 5 molar sulfuric acid (H_2_SO_4_) solution, leading to lower mechanical performance compared to the control samples [153]. On the other hand, in 2021, Yazdchi et al. evaluated the permeability of concrete after being subjected to freeze–thaw process at 300 cycles, with the addition of magnesium oxide (MgO) NPs. To evaluate the permeability, the sample was subjected to 5 bar for 24 h. As a result, they obtained an optimal percentage of 1% for a water/cement ratio of 0.44, causing a reduction in concrete permeability greater than 63% compared to the control. This was because the nanomaterial fills the nanopores of concrete, decreasing water penetration. Furthermore, by reducing the number of pores, more space is generated for the generation of hydration products, resulting in a decrease in permeability [154]. In 2022, Zhong et al. evaluated the effect of TiO_2_ NPs on water absorption in concrete made with recycled coarse aggregate subjected to freeze–thaw cycles. As a result, it was found that capillary water absorption increased with the increase in the percentage of recycled aggregate, while it decreased with the increase in the percentage of TiO_2_ NPs. After 150 freeze–thaw cycles, the porosity and cumulative water absorption decreased by 14.57% and 25.52%, respectively, when replacing 25% of recycled coarse aggregate and with the addition of 1.2% of TiO_2_ NPs by weight of the cement [155].

Table 3 presents additional studies in which different nanomaterials were incorporated into cementitious materials, evaluating their durability properties relative to a control sample. Results are expressed as percentages, where a positive sign (+) indicates improved performance compared to the control, and a negative sign (−) indicates a reduction in performance. Among the most common tests for assessing durability are chloride ion penetration resistance and water absorption resistance. A significant number of studies have utilized SiO_2_ nanoparticles, highlighting their positive contribution to the overall durability of cement-based materials.

### 3.3. Shrinkage

Shrinkage is a highly relevant phenomenon that naturally occurs when cementitious materials are applied in civil works. It refers to the volumetric reduction of these materials following placement, primarily due to water loss during the setting and hardening processes [166]. The evaporation of water from the cementitious mixture induces volume changes that generate internal stress. When the material has not yet developed sufficient mechanical strength, this can lead to the formation of microcracks [167]. Under mechanical loads, these microcracks may interconnect and propagate, resulting in macrocracks that compromise the structural integrity, increase permeability, and ultimately reduce the service life of the materials [168]. To mitigate this issue, various methods have been proposed, such as the use of steel mesh, polymeric microfibers, additives, or additions. Nevertheless, the problem of shrinkage remains significant. Recent research has demonstrated that the incorporation of nanomaterials into cementitious systems can effectively reduce shrinkage-related issues. For example, Abdullah et al. (2024) investigated the performance of concrete incorporating TiO_2_ NPs and varying percentages of rubber aggregates derived from recycled tires. Their study aimed to enhance different material properties. Results showed that increasing the amount of TiO_2_ NPs led to a reduction in drying shrinkage. However, the shrinkage values did not fall below those of the control sample, although they came close when the rubber aggregate content was reduced. The TiO_2_ NPs contributed to lower porosity and delayed crack formation, but the presence of rubber, due to its lower stiffness, resulted in higher deformation during the drying shrinkage process [132]. Similarly, Zhang et al. (2022) evaluated chemical shrinkage in cement pastes with varying dosages of SiO_2_ NPs. Their findings revealed that higher nanoparticle concentrations resulted in increased chemical shrinkage [169]. In contrast, Ikhlasi et al. (2023) studied drying shrinkage in concrete modified with different concentrations of pristine graphene NPs. The most favorable outcome was a 66% reduction in shrinkage, achieved with the addition of 0.1% graphene NPs. This improvement was attributed to the nanomaterial’s ability to refine the microstructure and its capacity for additional water absorption [170]. Table 4 summarizes findings from the literature regarding drying shrinkage in cementitious materials incorporating nanomaterials. The most used test to evaluate shrinkage was drying-induced cracking. In most cases, an increase in shrinkage was observed compared to the control, with values ranging from 3.5% to 68.18%. The table also reflects the use of nanomaterials across the three main types of cementitious materials: pastes, mortars, and concretes.

## 4. Nanotechnology Applied to Sustainable Cementitious Materials

Currently, global warming and extreme climate changes are critical issues that have caused significant transformations, negatively affecting the well-being of all living species on the planet. Among the various greenhouse gases contributing to climate change, carbon dioxide (CO_2_) is one of the most prominent [177].

The cement industry is one of the main industrial sources of CO_2_ emissions [178]. It is estimated that approximately 0.5 to 0.6 tons of CO_2_ are emitted for every ton of cement produced [179], representing nearly 7% of total global CO_2_ emissions [180]. In addition to emissions, the extraction of raw materials to produce cementitious materials poses a serious threat to ecosystems, disrupting both flora and fauna. These environmental challenges have prompted researchers to seek alternative strategies to reduce the negative environmental impacts of the construction industry. One such strategy is the use of recycled aggregates derived from construction and demolition waste. These have been studied with respect to different properties, replacement ratios, and performance outcomes [181,182,183,184]. However, several studies have shown that when recycled aggregates replace more than 30% of natural aggregates, the mechanical strength and durability of cementitious materials tend to decrease [185]. Therefore, an innovative alternative to enhance the performance of such materials involves the incorporation of nanomaterials into recycled cementitious composites [186,187,188]. The effectiveness of this approach depends on both the type and proportion of nanomaterial used. For instance, in 2024, Chinchillas et al. evaluated the incorporation of 0.1% polyvinylidene fluoride (PVDF) nanofibers (NFs) into mortar containing 25% recycled aggregate. They reported increases of 12% and 23% in compressive and flexural strength, respectively, along with a 3% reduction in porosity. These improvements were attributed to the excellent interaction between the nanomaterial and the hydration products, as well as the material’s ability to prevent crack formation at the nanoscale [189]. Similarly, in 2024, Deng et al. studied the effect of cellulose nanofibers on the interfacial transition zone (ITZ) of recycled concrete. Since the ITZ is often the weakest point in recycled cementitious composites, improving its performance is essential. Their results indicated that the proper incorporation of cellulose NFs (0.1%) significantly enhanced the nanostructure of this zone by promoting the formation of hydration product networks, thereby filling micropores in the matrix [190]. In another 2024 study, Al-Kheetan et al. evaluated the durability of concrete incorporating recycled aggregates and ZnO NPs. They replaced 30% and 50% of natural aggregates with recycled aggregates and added 0.5% ZnO NPs. The results showed a reduction in concrete porosity by 7% and 10% for the 30% and 50% recycled aggregate mixtures, respectively. In addition, water impermeability improved by 14.5% and 18%, respectively. These benefits were attributed to the reconfiguration of the porous structure induced by the nanoscale particles [191]. Additional studies on the integration of nanomaterials into recycled cementitious composites are summarized in Table 5. This table highlights the use of various recycled aggregate sources, including crushed concrete, masonry debris, and crumb rubber. Replacement ratios in these studies ranged from 25% to 100%.

The application of nanomaterials to recycled cementitious materials has generally resulted in improved mechanical and durability properties compared to reference mixes. Compressive strength was the most assessed mechanical property, with increases ranging from 12% to 46.7% relative to control samples. However, a common limitation reported was a reduction in workability. Durability was evaluated through various tests, including porosity, chloride ion permeability, water absorption, and freeze–thaw resistance. In most cases, results showed improvements, indicating that the incorporation of nanomaterials enhances the performance of recycled cementitious composites.

## 5. Conclusions

This article presents a comprehensive literature review on the current state of synthesis methods for various nanomaterials (NPs and NFs) and their application in enhancing both conventional and recycled cementitious materials as a contribution to the construction industry.

The various synthesis methods for nanomaterials (NPs and NFs) have proven to be highly efficient and promising for large-scale production; they enable the generation of nanomaterials with tailored sizes, shapes, and specific properties. These synthesis strategies allow for the integration of nanoscale components into cementitious and recycled materials to enhance their mechanical performance and durability. The application of nanotechnology to cement and mortar composites has gained significant relevance and made considerable progress in recent years. However, an analysis of the most recent publications reveals a high variability in results, which can be attributed to differences in synthesis parameters as well as variations in the type, size, concentration, and morphology of the nanomaterials used.

The studies reviewed in this article demonstrate that the incorporation of different nanomaterials into cement-based materials, such as concrete and mortar, offers several benefits. These include improvements in compressive and flexural strength, reduction in porosity, decreased water permeability, reinforcement of the cement matrix, enhanced cement hydration, and reductions in shrinkage and cracking, among others. Nevertheless, it is important to emphasize that further awareness and deeper investigation are still required, as several challenges remain to be addressed. To enable the practical use of nanomaterials in the construction industry, it is essential to develop tools and processes that allow for large-scale nanomaterial production while minimizing cost, time, and environmental impact. Another significant limitation identified in this review is the need to establish highly specific methodologies for achieving homogeneous dispersion of nanomaterials and determining optimal dosage levels. Improper dispersion can lead to particle agglomeration and structural deficiencies. Therefore, future research should adopt a predictive approach, where computational models guide experimental designs by estimating optimal nanoparticle dosages, the most effective dispersion/separation techniques, and the most scalable synthesis methods. These aspects are essential to making the integration of nanotechnology into the construction industry a tangible reality, thus enabling its widespread adoption worldwide.

Based on this review, we can conclude that nanotechnology and nanomaterials represent powerful tools for advancing cementitious materials, thus contributing to the development of sustainable, resilient, and smart cities and communities. Their application reflects the synergy of innovation and sustainability, aimed at protecting limited resources while driving technological evolution in construction.

## Figures and Tables

**Table 1:** Classification of nanomaterials (Information obtained by Barhoum et al., 2022 [14]).

Classification Parameter	Classification	Examples
Dimension	0D	Nanoparticles
	1D	Nanofibers, nanotubes
	2D	Graphene, phosphorene
	3D	Bulk solids, Nanostructures films
Phase composition	Single-phase solids	Sigle metal or oxide nanoparticles
	Multi-phase solids	Core shell
Origin	Engineered	“Top–down” and “bottom–up” methods
	Incidental	Storm, cosmic dust, volcanic activity
	Natural	Micro and higher organisms
Composition	Carbonaceous	All allotropic forms
	Organic	Molecular self-assembled
	Inorganic	Metal nanoparticles, QD
	Composites	Bimetallic, core–shell
Dispersion	Dispersed nanomaterials	Isometric, inhomogeneous
	Aggregated nanomaterials	Isometric, inhomogeneous

**Table 3:** Durability properties of cementitious materials with the addition of nanomaterials.

Year	Nanomaterial	Test	Results	Reference
2021	Al_2_O_3_ NPs (3%) in concrete	Resistance ion Cl^−^	+14.5%	[156]
2021	SiO_2_ NPs (0.5%) in concrete	Electrical resistivity	+61.5%	[156]
2021	Al_2_O_3_ NPs (1%) in concrete	Water absorption	−6%	[156]
2021	Carbon nanotubes (0.01%) + Nano clay (5%) in concrete	Water penetration test	+150%	[157]
2021	Carbon Nanotubes (0.01%) in concrete	Resistance ion Cl^−^	+82.3%	[157]
2021	SiO_2_ NPs (1%) in concrete	Sorptivity	−84.8%	[158]
2021	SiO_2_ NPs (1%) + Carbon Nanotubes (0.3%) in mortar	Length expansion	−14.3%	[159]
2021	SiO_2_ NPs (1%) + Carbon Nanotubes (0.3%) in mortar	Abrasion	−29.2%	[159]
2021	SiO_2_ NPs (1%) + Carbon Nanotubes (0.3%) in mortar	Resistivity	+5.1%	[159]
2021	SiO_2_ NPs (1%) + Carbon Nanotubes (0.3%) in mortar	Ultrasonic pulse velocity	+25%	[159]
2021	SiO_2_ NPs (2%) in concrete	Resistance ion Cl^−^	−14.7%	[160]
2021	GO + Al_2_O_3_ NPs (2%)	Compressive strength under freeze–thaw	−10.8%	[161]
2021	Al_2_O_3_ NPs (2%)	Compressive strength under freeze–thaw	−12.9%	[161]
2023	TiO_2_ NPs (2.5%) in concrete	Corrosion rate (6 months)	−86.5%	[162]
2023	PVA fibers (2%) + Graphene Oxide NPs (0.05%) in mortar	pH test	−8.3%	[163]
2023	PVA fibers (2%) + Graphene Oxide NPs (0.05%) in mortar	Resistance ion Cl^−^	−17.3%	[163]
2023	Graphene nanoplatelet (0.05%) + Sodium polyacrylate (0.11%)	Chloride ion penetration depth under freeze–thaw	−42%	[164]
2024	Al_2_O_3_ NPs (1%) + PVA fibers (0.3%) in mortar	Resistance ion Cl^−^	−35.9%	[152]
2024	Graphene oxide NPs (Surface treatment with 31.5 µg cm^−2^) in concrete	Water absorption by capillary action.	−21.4%	[165]
2024	Graphene oxide NPs (Surface treatment with 31.5 µg cm^−2^) in concrete	Water absorption by immersion.	−41.8%	[165]
2024	TiO_2_ NPs (2%) + Crumb rubber aggregate (10%) in concrete	Sorptivity	−25.5%	[132]
2024	TiO_2_ NPs (2%) + Crumb rubber aggregate (10%) in concrete	Apparent Porosity	−37.8%	[132]

Note: In the results, the + symbol corresponds to an increase relative to the control, while the − symbol represents a decrease.

**Table 4:** Shrinkage of cementitious materials with the addition of nanomaterials.

Year	Nanomaterial (%)	Test	Results	Reference
2020	SiO_2_ NPs (1.2%) in cement paste	Chemical shrinkage	+57.5%	[171]
2021	SiO_2_ NPs (3%) in concrete	Drying	+13.3%	[156]
2022	SiO_2_ NPs (0.4%) in cement paste	Chemical Shrinkage	+3.5%	[169]
2023	Pristine Graphene NPs (0.1%) in concrete	Drying shrinkage	−66%	[170]
2023	Graphene oxide NPs (0.01%) in concrete	Drying shrinkage	+1.3%	[172]
2024	Graphene oxide NPs (0.08%) + fly ash (15%) in concrete	Drying shrinkage	−45.7%	[173]
2024	TiO_2_ NPs (2%) + Crumb rubber aggregate (10%) in concrete	Drying shrinkage	+12.5%	[132]
2025	TiO_2_ NPs (8%) in mortar	Drying shrinkage	+68.18%	[174]
2025	SiO_2_ NPs (1.5%) in concrete	Autogenous shrinkage	+37.87%	[175]
2025	Carbon nanotubes (0.2%) + Rice husk ash (15%) in concrete	Drying shrinkage	−5.04%	[176]

Note: The results are shown based on a control sample, where the + symbol corresponds to an increase in the result relative to the control, while the − symbol represents a decrease.

**Table 5:** Research on the incorporation of nanomaterials into recycled cementitious materials.

Year	Nanomaterial (%)	Recycled Aggregate	Test	Results	Reference
2022	Carbon NFs (0.25%) + Steel Fibers (2%) in concrete	Recycled coarse aggregate (100%)	Compressive strength	Increase 23.1%, but decrease workability	[192]
2024	PVDF NFs (0.1%) in mortar	Recycled fine aggregate (25%)	Compressive strength	Increase 12%	[189]
2024	PVDF NFs (0.1%) in mortar	Recycled fine aggregate (25%)	Flexural strength	Increase 23%	[189]
2024	PVDF NFs (0.1%) in mortar	Recycled fine aggregate (25%)	Porosity	Decrease 3%	[189]
2024	PVDF NFs (0.1%) in mortar	Recycled fine aggregate (25%)	Chloride ion permeability	Increase 20%	[189]
2023	SiO_2_ NPs (1%) in concrete	Recycled fine aggregate (25%)	Compressive strength	Increase 14%	[193]
2023	Al_2_O_3_ NPs (1%) in concrete	Brick aggregate (30%)	Water absorption	Decrease 24.2%	[194]
2023	SiO_2_ NPs (1%) in concrete	Brick aggregate (30%)	Water absorption	Decrease 27.9%	[194]
2023	Al_2_O_3_ NPs (2%) in concrete	Brick aggregate (30%)	Compressive strength	Increase 43.4%	[194]
2023	SiO_2_ NPs (2%) in concrete	Brick aggregate (30%)	Compressive strength	Increase 33.1%	[194]
2023	SiO_2_ NPs (4%) in concrete	Recycled coarse aggregate (30%)	Dry wet cycle	Decrease 9.4%	[195]
2024	SiO_2_ NPs (3%) in concrete	Recycled coarse aggregate (100%)	Compressive strength	Increase 20.6%	[196]
2024	SiO_2_ NPs (3%) in concrete	Recycled coarse aggregate (100%)	Split Tensile Strength	Increase 23.8%	[196]
2024	SiO_2_ NPs (3%) in concrete	Recycled coarse aggregate (100%)	Bond Strength	Increase 20.92%	[196]
2022	TiO_2_ NPs (1.2%) in concrete	Recycled coarse aggregate (25%)	Freeze–thaw	Decrease 25.52% water absorption and 14.57% porosity	[155]
2024	TiO_2_ NPs (2%) in concrete	Crumb rubber concrete (10%)	Sorptivity	Decrease 25.5%	[132]
2024	TiO_2_ NPs (2%) in concrete	Crumb rubber concrete (10%)	Apparent Porosity	Decrease 37.8%	[132]
2023	Pristine graphene (0.2%) in concrete	Recycled coarse aggregate (50%)	Workability	Increase 13%	[197]
2023	Pristine graphene (0.2%) in concrete	Recycled coarse aggregate (50%)	Compressive strength	Increase 21%	[197]
2023	Pristine graphene (0.2%) in concrete	Recycled coarse aggregate (50%)	Tensile strength	Increase 12%	[197]
2023	Pristine graphene (0.2%) in concrete	Recycled coarse aggregate (50%)	Water absorption	Decrease 22%	[197]
2023	Pristine graphene (0.2%) in concrete	Recycled coarse aggregate (50%)	Drying shrinkage	Decrease 20%	[197]
2022	Carbon NTs (0.1%) in concrete	Recycled coarse aggregate (50%)	Compressive strength	Increase 46.7%	[198]

## Data Availability

No new data were created or analyzed in this study. Data sharing is not applicable to this article.
